# Interleukin‐8 Overexpressing Collagen Microgel‐Based Cellular Microtissue Accelerates the Healing of Diabetic Foot Ulcers

**DOI:** 10.1002/smll.202511027

**Published:** 2025-11-26

**Authors:** Haeun Chung, Won Young Jang, Jung‐Kyun Choi, Sang‐Heon Kim

**Affiliations:** ^1^ Center for Biomaterials Biomedical Research Institute Korea Institute of Science and Technology (KIST) Seoul 02792 Republic of Korea; ^2^ Division of Bio‐Medical Science and Technology KIST School Korea University of Science and Technology (UST) Seoul 02792 Republic of Korea

**Keywords:** 3D cell culture, collagen microgel, diabetic foot ulcer, interleukin‐8, regenerative medicine, stem cell therapy, wound healing

## Abstract

Diabetic foot ulcers (DFUs) represent a major clinical challenge due to impaired healing and limited therapeutic options. Although stem cell therapy offers regenerative potential, its efficacy is restricted by poor survival and engraftment at the injury site. To address this, collagen microgels (CMGs) are developed to assemble with cells to form CMG‐based cellular microtissues (CCMs) with enhanced porosity, mass transfer, and viability. However, the diabetic microenvironment impairs healing by suppressing angiogenesis, keratinocyte migration, and fibroblast proliferation. Transcriptomic profiling identifies interleukin‐8 (IL‐8) as a key factor with multifaceted roles in promoting angiogenesis, migration, and proliferation. Compared with conventional aggregates, CCMs enhance adhesion, prevent anoikis, improve survival, and upregulate IL‐8 via FGFR‐integrin‐ERK signaling. Functional studies using shRNA knockdown and adenoviral overexpression validate the therapeutic role of IL‐8. In vitro co‐culture with keratinocytes, fibroblasts, and endothelial cells shows that CCMs promoted migration, proliferation, and angiogenesis–effects diminished by IL‐8 knockdown and amplified by overexpression. In a rat DFU model, CCMs accelerate wound closure by enhancing granulation tissue formation, collagen deposition, and expression of proliferative and angiogenic markers, all modulated by IL‐8. These findings establish CCMs as an effective therapeutic platform for DFUs and highlight IL‐8 overexpression as a strategy to further potentiate regeneration.

## Graphical abstract

Collagen microgels (CMGs) incubated with cells self‐assemble into CMG‐based cellular microtissues (CCMs), enhancing cell adhesion and upregulating IL‐8 expression. A combinatorial strategy that integrates the CMG platform with IL‐8 overexpression promotes cell proliferation, migration, angiogenesis, and survival, thereby accelerating diabetic wound healing. Illustration is created using Biorender.com.

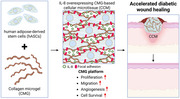

## 1 Introduction

Diabetes is a globally prevalent metabolic disorder, affecting ≈830 million individuals worldwide as of 2022.^[^ ^1^ ^]^ Among its complications, diabetic foot ulcers (DFUs) represent one of the most severe, affecting ≈25% of diabetic patients.^[^ ^2^ ^]^ Clinically, DFUs are characterized by full‐thickness wounds on the foot that can progressively extend into underlying soft tissues and bone if inadequately managed, potentially leading to limb amputation and even death.^[^ ^3^ ^]^ Notably, DFUs account for 50–70% of all nontraumatic lower‐limb amputations, with one leg being amputated worldwide every 30 s.^[^ ^4^ ^]^ Despite their prevalence and severity, current DFU treatments primarily rely on conventional wound care approaches such as debridement, off‐loading, medication, dressing, and infection control.^[^ ^5^ ^]^ However, these methods often fail to achieve satisfactory healing, leading to prolonged treatment duration, increased amputation risk, and high morbidity and mortality.^[^ ^5^ ^]^ Consequently, there is an urgent need for more effective and durable therapeutic strategies for DFUs.

Recent advances have explored novel therapies involving growth factors, microRNAs, pharmacological agents, biomaterials, and stem cell‐based approaches.^[^ ^6^, ^7^, ^8^ ^]^ Among these, mesenchymal stem cells (MSCs) have gained particular interest due to their robust paracrine activity, characterized by the secretion of trophic factors that promote granulation tissue formation, collagen deposition, and angiogenesis.^[^ ^9^, ^10^ ^]^ Nevertheless, the therapeutic efficacy of MSCs in chronic wound healing remains limited, primarily due to poor cell survival and engraftment within the hostile diabetic microenvironment characterized by hypoxia, oxidative stress, and chronic inflammation.^[^ ^11^, ^12^ ^]^ Because MSCs exert their regenerative effects predominantly via paracrine signaling, sustaining cell viability post‐transplantation is essential for prolonged therapeutic activity.^[^ ^13^ ^]^

To enhance MSC survival, various biomaterial scaffolds mimicking the extracellular matrix (ECM) have been developed. Among these, collagen‐based hydrogels and scaffolds have been widely used for their biocompatibility and ability to provide a supportive 3D microenvironment that enhances cell adhesion, survival, and function.^[^ ^12^ ^]^ In particular, microgel platforms offer several advantages, including injectability, uniform cell distribution, increased porosity, and facilitation of host cell infiltration.^[^ ^14^, ^15^ ^]^ Cells pre‐attached to microgels receive mechanical support post‐transplantation, reducing anoikis and enhancing therapeutic retention. We previously developed a collagen microgel (CMG) platform composed of collagen and hyaluronic acid, which demonstrated improved post‐transplantation cell survival and increased angiogenic potential due to enhanced mass transfer through its porous structure.^[^ ^16^ ^]^ The natural ECM composition and preclinical efficacy of collagen underscore CMG as a promising scaffold for stem cell delivery.

Wound healing is a complex and coordinated process involving hemostasis, inflammation, proliferation, and remodeling. In DFUs, this sequence is profoundly disrupted by diabetic pathologies, including chronic hyperglycemia, ischemia, hypoxia, inflammation, and neuropathy.^[^ ^17^ ^]^ These conditions impair angiogenesis and inhibit keratinocyte and fibroblast migration and proliferation, resulting in delayed wound healing.^[^ ^18^, ^19^ ^]^ Notably, Kruse et al. reported that local hyperglycemia directly impairs keratinocyte and fibroblast migration, thereby delaying healing.^[^ ^20^ ^]^ To address these deficiencies, we identified interleukin‐8 (IL‐8) as a promising therapeutic target due to its pro‐angiogenic, migratory, and proliferative properties—functions that are diminished in diabetic wounds. IL‐8 has been shown to promote keratinocyte proliferation and migration, as well as rapid granulation tissue formation, highlighting its therapeutic relevance.^[^ ^21^, ^22^ ^]^ Although the CMG platform alone enhanced cell viability and transiently increased IL‐8 expression, its secretion declined over time, potentially compromising therapeutic efficacy.^[^ ^16^ ^]^

IL‐8 is a cytokine regulated by a variety of biochemical and biophysical cues. Hypoxia‐inducible factor 1‐alpha (HIF1α) and 3D formation have been identified as significant regulators of IL‐8 expression, particularly in cancer models.^[^ ^23^, ^24^, ^25^ ^]^ Engagement of integrins with ECM components such as collagen IV and fibronectin activates outside‐in signaling cascades involving integrin beta 1 (ITGB1), focal adhesion kinase (FAK), and extracellular response kinase 1/2 (ERK1/2), leading to cytoskeletal reorganization and subsequent IL‐8 upregulation.^[^ ^26^, ^27^ ^]^ In parallel, receptor tyrosine kinases, including fibroblast growth factor receptors (FGFRs), have been shown to enhance IL‐8 expression via canonical mitogen‐activated protein kinase (MAPK) and phosphoinositide 3‐kinase (PI3K) pathways. Previous studies reported that FGFR1 activation upregulates IL‐8 through c‐Jun N‐terminal kinase (JNK) or ERK signaling.^[^ ^28^, ^29^ ^]^ Furthermore, crosstalk between integrins and FGFRs has been shown to enhance IL‐8 expression.^[^ ^29^, ^30^ ^]^ Based on this, we hypothesized that our CMG platform would not only improve cell survival but also enhance IL‐8 expression, thereby contributing to improved therapeutic efficacy in DFU treatment.

IL‐8 is a promising therapeutic target in wound healing. While primarily recognized as a chemoattractant for neutrophils, IL‐8 also acts on fibroblasts, keratinocytes, and endothelial cells to induce chemotaxis toward the injury site.^[^ ^31^ ^]^ In keratinocytes, IL‐8 directly stimulates migration, accelerating wound gap closure and re‐epithelialization.^[^ ^32^ ^]^ In fibroblasts, IL‐8 promotes both migration and proliferation, contributing to granulation tissue formation.^[^ ^33^ ^]^ Moreover, angiogenesis is a critical process in tissue repair, and IL‐8 functions as a potent angiogenic factor by enhancing endothelial cell migration, proliferation, and survival, ultimately facilitating neovascularization.^[^ ^34^ ^]^ Given its multifunctional roles in promoting migration, proliferation, and angiogenesis—key processes in wound healing—IL‐8 represents a compelling therapeutic target for diabetic wounds.

In this study, we sought to combine sustained IL‐8 overexpression with our CMG‐based stem cell delivery platform to overcome the limitations of conventional MSC therapy. Human adipose‐derived stem cells (hASCs) were cultured with CMGs to generate CMG‐based cellular microtissues (CCMs). To validate the therapeutic role of IL‐8 in DFUs, we modulated IL‐8 expression through short hairpin RNA (shRNA)‐mediated knockdown and adenoviral overexpression. Recombinant adenovirus‐mediated overexpression maintained elevated IL‐8 levels and maximized therapeutic outcomes—significantly enhancing granulation tissue formation, collagen deposition, and expression of angiogenic and proliferative markers in a rat DFU model. Collectively, our findings demonstrate that IL‐8‐overexpressing CCMs represent a promising strategy to enhance stem cell‐based therapy for DFUs.

## 2 Results

### 2.1 Cell‐to‐CMG Ratio on Adhesion, Viability, and Gene Expression

**Figure 1:**
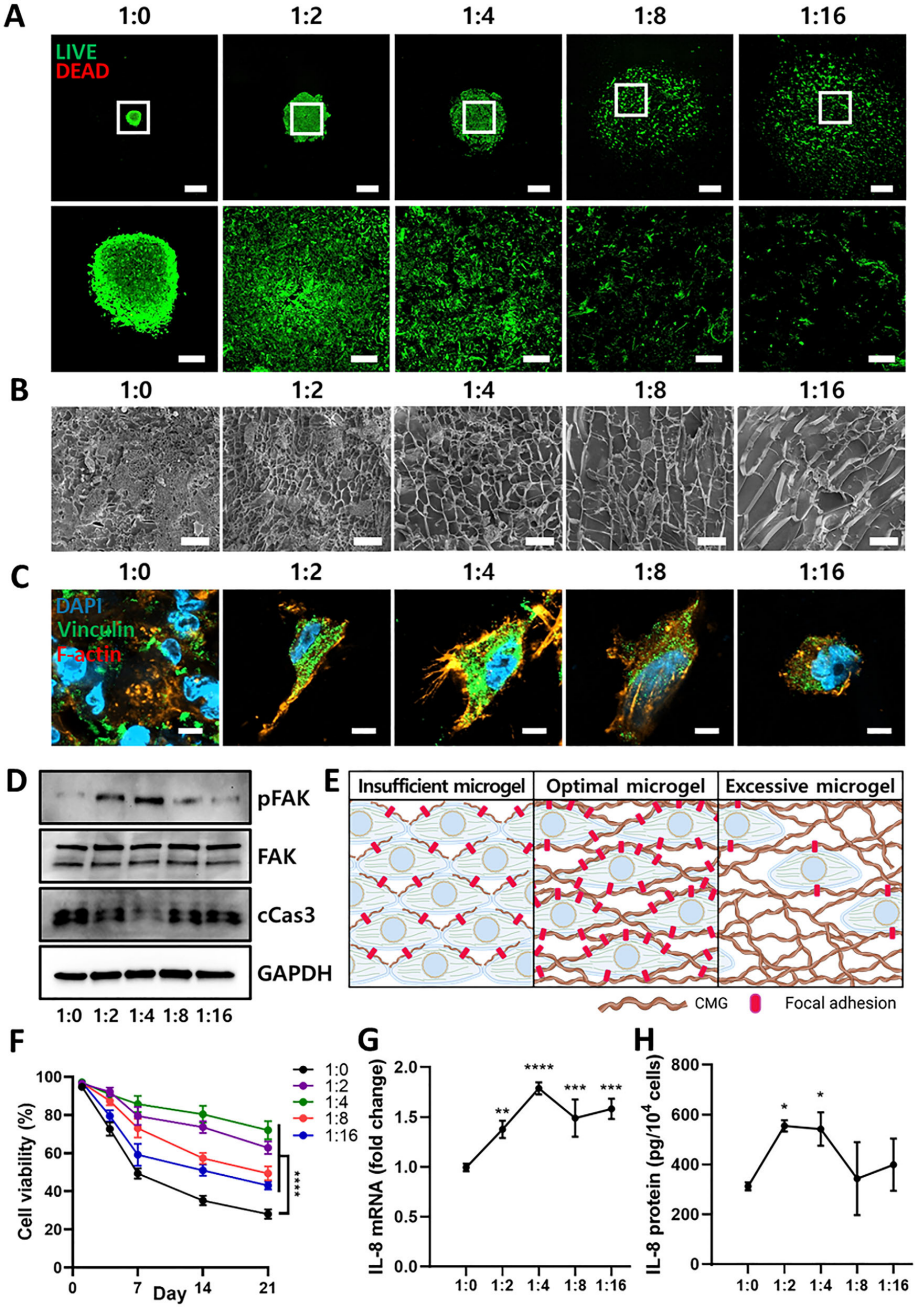
Effect of varying cell:CMG ratios on cell adhesion, viability, and gene expression. A) Representative LIVE/DEAD fluorescence images of 3D cell constructs formed at varying cell:CMG ratios (1:0, 1:2, 1:4, 1:8, and 1:16; 2 × 10^5^ hASCs/sample, ratio determined by pellet size) after 1 day of culture. Scale bars: 1 mm; inset: 200 µm. B) Representative cryo‐SEM images of 3D cell constructs showing internal microstructure. Scale bar: 20 µm. C) Representative fluorescent images showing staining of vinculin (green) and F‐actin (red), visualized using an LSM800 confocal microscope. Nuclei were counterstained with DAPI. Scale bar: 5 µm. D) Representative western blot images of cell lysates obtained from 3D cell constructs after 3 days of culture. GAPDH was used as a loading control. E) Schematic illustration depicting the influence of microgel density relative to cell density on cell adhesion. Both insufficient and excessive microgel densities restrict cell adhesion, whereas optimal density promotes FA formation and enhances cell adhesion. Illustration was created using Biorender.com. F) Quantification of cell viability in 3D cell constructs over 21 days, determined by the trypan blue exclusion assay (*n* = 3 per group). Two‐way ANOVA; ^****^ *p* < 0.0001 compared with the 1:0 group. G,H) Line curves showing the relationship between 3D cell constructs formed at different cell:CMG ratios and IL‐8 expression at the mRNA (G) and protein (H) levels (*n* = 3 per group). One‐way ANOVA; ^*^ *p* < 0.05, ^**^ *p* < 0.01, ^***^ *p* < 0.001, ^****^ *p* < 0.0001 compared to the 1:0 group. All data are presented as mean ± SD.

To investigate the influence of the cell:CMG ratio on 3D formation, hASCs were cultured with CMG at pellet volume ratios ranging from 1:0 to 1:16. CMGs alone failed to form aggregates, whereas co‐culture with hASCs induced aggregation, with the extent of contraction inversely correlated with CMG content (Figure S1A, Supporting Information). Biocompatibility was confirmed via LIVE/DEAD staining and trypan blue exclusion assays, which revealed no cytotoxicity after one day of culture (**Figure** **1**A; Figure S1C, Supporting Information). Cryo‐scanning electron microscopy (cryo‐SEM) analysis revealed that the average pore size increased with higher cell:CMG ratios, ranging from 2.7 ± 1.2 at 1:0 to 13.3 ± 3.2 µm at 1:16 (Figure 1B; Figure S1D–J, Supporting Information). Porosity increased correspondingly from 10.0 ± 3.8 % to 66.2 ± 11.6 %. These results indicate that the scaffold microarchitecture is governed by the relative densities of cells and microgels.

To examine how the cell:CMG ratio modulates cell adhesion and viability, we assessed focal adhesion (FA) formation, apoptosis, mechanical properties, and gene expression. Immunofluorescence staining for vinculin revealed a peak in FA area at a 1:4 ratio, which decreased at higher ratios (1:8 and 1:16), indicating that optimal cell‐matrix interactions occur at this intermediate density (Figure 1C; Figure S1E, Supporting Information). Consistently, western blot analysis showed maximal levels of pFAK and reduced levels of cleaved caspase‐3 at the 1:4 ratio, suggesting enhanced adhesion and suppressed apoptosis (Figure 1D). A long‐term cell viability assay over 21 days further supported these findings, showing that hASCs cultured without CMG (1:0) experienced the most rapid decline in viability, whereas the 1:4 ratio maintained the highest viability. Higher cell:CMG ratios (1:8 and 1:16) resulted in reduced viability (Figure 1F). Rheological analysis corroborated these findings, as both the elastic and viscous moduli were highest at the 1:4 ratio, reflecting improved structural integrity (Figure S1B, Supporting Information). As illustrated in Figure 1E, these results indicate that an optimal cell:CMG balance is essential for robust adhesion and favorable mechanical properties, whereas insufficient or excessive CMG impairs adhesion.

We next examined the effect of varying cell:CMG ratios on IL‐8 expression. IL‐8 levels peaked at a 1:4 ratio at both the mRNA and protein levels, highlighting the influence of matrix density on gene expression (Figure 1G,H). Collectively, these findings demonstrate that the 1:4 ratio provides the most favorable balance of microarchitecture, adhesion, viability, and paracrine signaling. Accordingly, this condition was designated as the CMG‐based cellular microtissue (CCM), while the 1:0 group served as the cell aggregate (CA) control in subsequent experiments.

### 2.2 Mechanism of IL‐8 Regulation in CMG‐Based Cellular Microtissues

**Figure 2:**
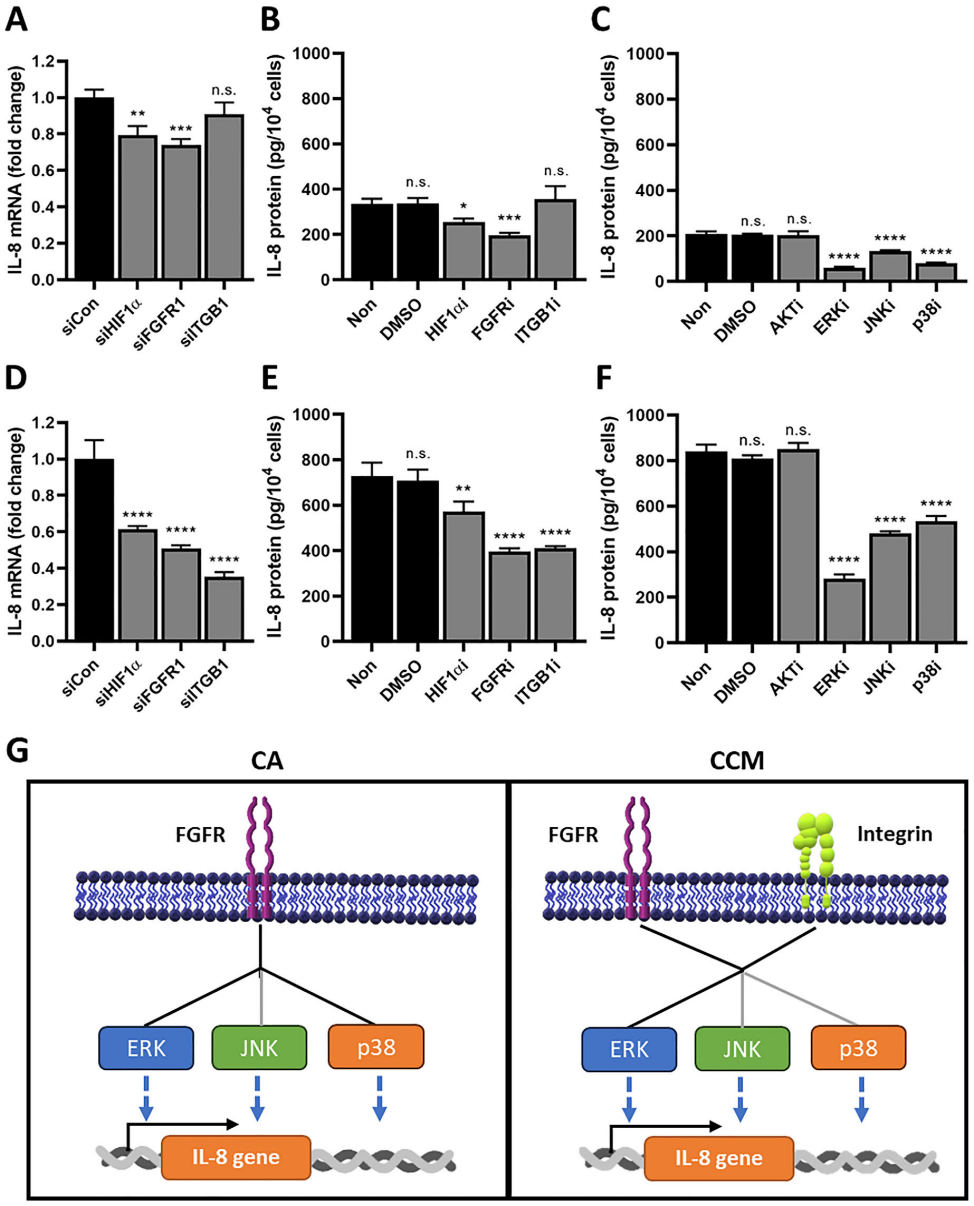
CMG‐mediated regulation of IL‐8 expression. A,D) IL‐8 mRNA levels in CA (A) and CCM (D) after treatment with scrambled siRNA (siCon) or siRNA targeting HIF1α, FGFR1, or ITGB1 at 10 nm, as determined by RT‐PCR (*n* = 3 per group). One‐way ANOVA; n.s., not significant; ^**^ *p* < 0.01, ^***^ *p* < 0.001, ^****^ *p* < 0.0001 compared with the siCon group. B,E) IL‐8 protein levels in CA (B) and CCM (E) after treatment with chemical inhibitors or a function‐blocking antibody targeting HIF1α, FGFR1, or ITGB1 at predetermined concentrations of 20 µm, 40 nm, and 5 µg mL^−1^, respectively (*n* = 3 per group). One‐way ANOVA; n.s.; not significant, ^*^ *p* < 0.05, ^**^ *p* < 0.01, ^***^ *p* < 0.001, ^****^ *p* < 0.0001 compared with the Non group. (C,F) IL‐8 protein levels in CA C) and CCM F) after treatment with chemical inhibitors targeting Akt, ERK, JNK, and p38 at 10 µm, as determined by ELISA (*n* = 3 per group). One‐way ANOVA; n.s., not significant; ^****^ *p* < 0.0001 compared with the Non group. H) Schematic illustration of the signaling pathways regulating IL‐8 expression in CA and CCM. Black and gray lines indicate major and minor pathways, respectively. All data are presented as mean ± SD.

IL‐8 expression is known to be regulated by hypoxia, FGFR, and integrin‐mediated signaling. To elucidate the mechanisms underlying elevated IL‐8 levels in CCMs, we employed siRNAs, pharmacological inhibitors, and function‐blocking antibodies targeting FGFR, HIF1α, and ITGB1. Optimal concentrations and exposure times were predetermined (Figure S2, Supporting Information). In the CA group, inhibition of FGFR and HIF1α significantly decreased IL‐8 mRNA and protein expression, with FGFR blockade producing a more pronounced effect (**Figure** **2**A,B). In the CCM group, inhibition of FGFR, HIF1α, or ITGB1 each led to decreased IL‐8 levels, with FGFR and ITGB1 suppression exerting the strongest effects (Figure 2D,E). These results suggest that FGFR and integrin signaling play dominant roles in IL‐8 regulation under CCM conditions, with hypoxia also contributing.

To identify the downstream pathways involved, cells were treated with inhibitors targeting Akt, ERK, JNK, and p38. IL‐8 expression was unaffected by Akt inhibitor in both CA and CCM groups, but ERK, JNK, and p38 inhibition each reduced IL‐8 levels (Figure 2C,F). In the CA group, ERK and p38 served as the primary mediators, while in CCMs, IL‐8 expression was most strongly suppressed by ERK inhibition, identifying ERK as the predominant downstream mediator (Figure 2G).

### 2.3 In vitro Evaluation of IL‐8 on Angiogenic, Migratory, and Proliferative Functions

IL‐8 knockdown and overexpression were achieved by transfection with shRNA targeting IL‐8 (shIL‐8) and transduction with recombinant adenovirus encoding IL‐8 (rAd‐IL‐8), respectively. shIL‐8 reduced IL‐8 expression by ≈50%, whereas rAd‐IL‐8 induced approximately a twofold increase relative to their respective controls. These effects persisted for ≈14 and 35 days, respectively (Figure S3A–J, Supporting Information).

**Figure 3:**
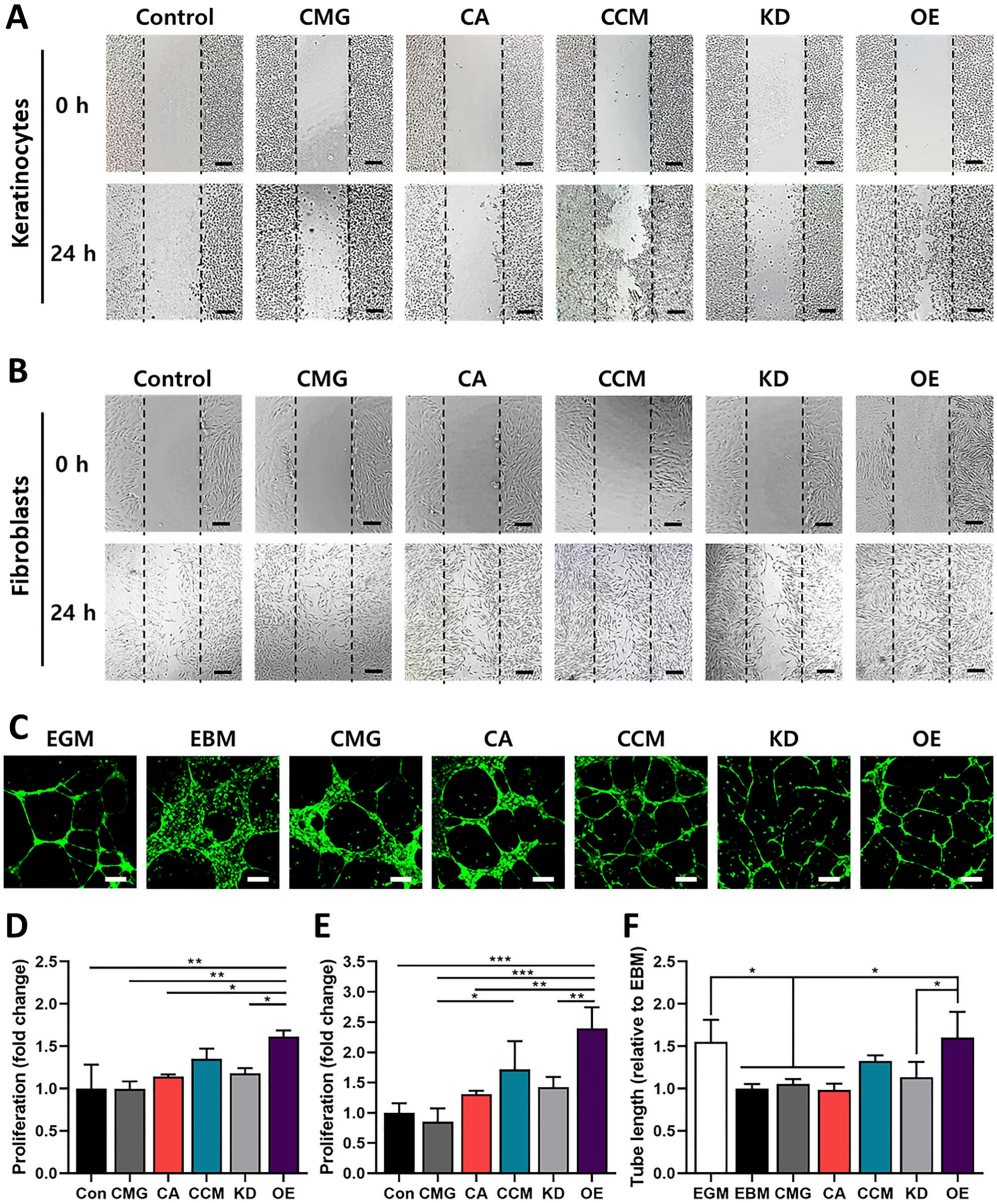
Evaluation of the migratory, angiogenic, and proliferative properties of IL‐8. A,B) Representative bright‐field images showing the migration of HaCaT keratinocytes (A) and human dermal fibroblasts (B) into the scratch area after co‐culture with control, CMG, CA, CCM, KD, or OE at 0 and 24 h post‐scratch. Scale bars: 100 µm. C) Representative fluorescence images showing tube formation of GFP‐tagged HUVECs after 24 h co‐culture with EBM, EGM, CMG, CA, CCM, OE, or KD groups. Scale bars: 200 µm. D,E) Quantification of the proliferation of keratinocytes (D) and fibroblasts (E) at 24 h after co‐culture (*n* = 3 per group). One‐way ANOVA; ^*^ *p* < 0.05, ^**^ *p* < 0.01, ^***^ *p* < 0.001. F) Quantification of HUVEC tube length after 24 h co‐culture with each group (*n* = 3 per group). One‐way ANOVA; ^*^ *p* < 0.05. All data are presented as mean ± SD.

Scratch assays demonstrated that co‐culture with CCMs enhanced keratinocyte and fibroblast migration compared with the controls (**Figure** **3**A,B; Figure S4, Supporting Information). Migration was significantly reduced in IL‐8 knockdown CCMs (KD) and enhanced in IL‐8 overexpressing CCMs (OE), confirming the pro‐migratory role of IL‐8. Similarly, proliferation assays revealed reduced keratinocyte and fibroblast proliferation in the KD group and increased proliferation in the OE group, as determined by DNA quantification (Figure 3D,E). To assess angiogenic potential, human umbilical vein endothelial cells (HUVECs) were co‐cultured with each group, and tube formation was quantitatively analyzed. Co‐culture with CCMs moderately enhanced tube formation compared with the controls, whereas this effect was attenuated in the KD group and further potentiated in the OE group (Figure 3C,F). Collectively, these findings demonstrate that IL‐8 facilitates cell migration, proliferation, and angiogenesis in vitro.

### 2.4 Therapeutic Effects of IL‐8 Expressing CCMs in Rat DFU Model

**Figure 4:**
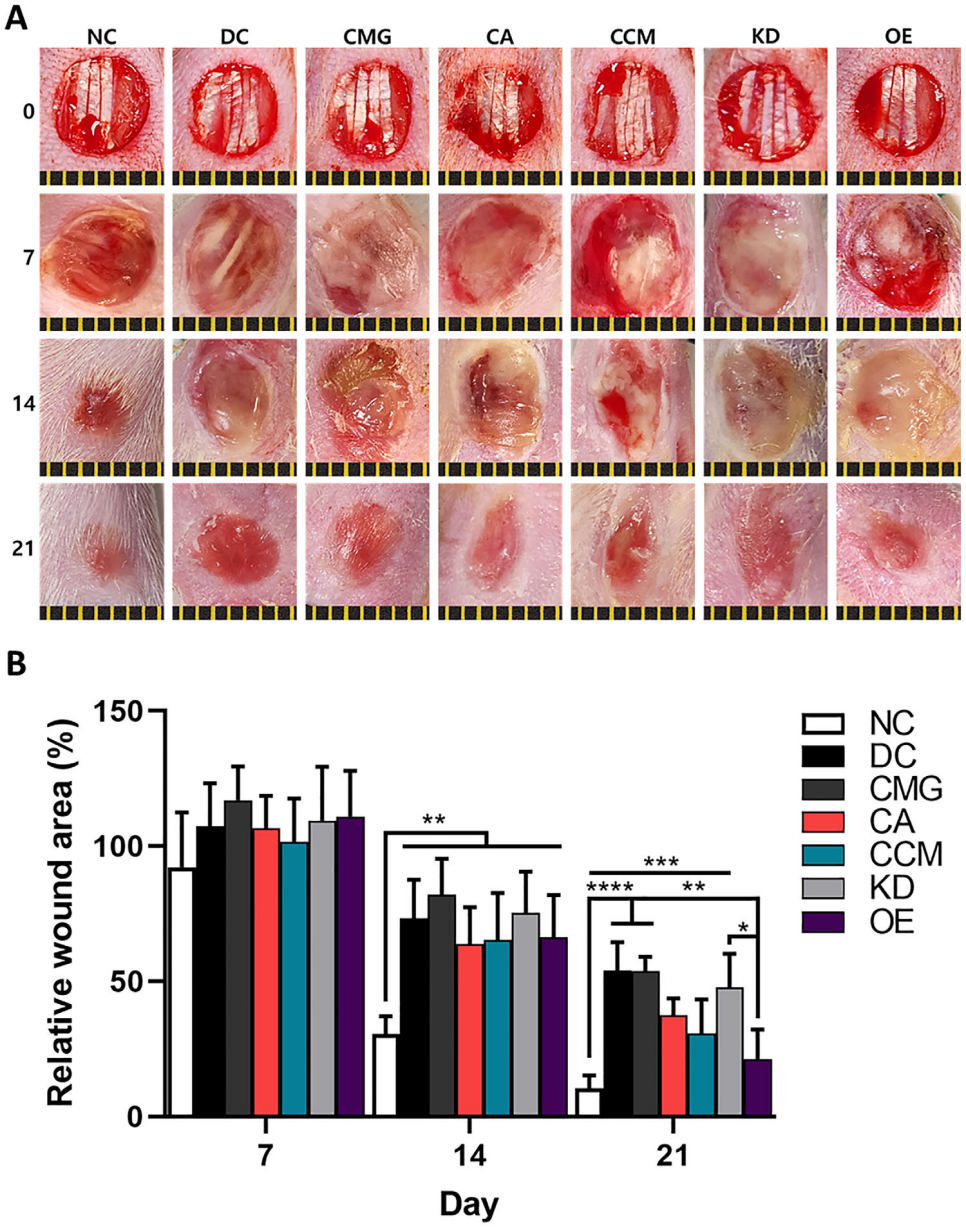
Effect of IL‐8‐expressing CCM on wound healing in rat DFU model. A,B) Representative photographs (A) and quantification of wound closure rate (B) in the rat DFU model over 21 days (*n* = 5/group). Diabetes was induced by a single intraperitoneal injection of 70 mg kg^−1^ of streptozotocin in male Sprague‐Dawley rats, and diabetic status was confirmed when blood glucose levels exceeded 300 mg dL^−1^ one week after induction. A 6 mm full‐thickness wound was created on the dorsal hindlimb, and non‐diabetic (NC) and diabetic rats were treated with control (DC), CMG, CA, CCM, KD, or OE at a dose of 4 × 10^5^ hASCs/wound. Scale bar: 1 mm. All data are presented as mean ± SD. One‐way ANOVA; ^*^ *p* < 0.05, ^**^ *p* < 0.01, ^***^ *p* < 0.001, ^****^ *p* < 0.0001.

In the rat DFU model, wound area analysis revealed significantly accelerated healing in the non‐diabetic control (NC) group compared with the diabetic control (DC) group, confirming the detrimental effect of the diabetic microenvironment (**Figure** **4**A,B). Treatment with CCMs modestly improved wound closure, whereas IL‐8 knockdown impaired healing, and IL‐8 overexpression markedly enhanced closure relative to the DC group. These results indicate that IL‐8 plays a crucial role in promoting wound repair under diabetic conditions.

**Figure 5:**
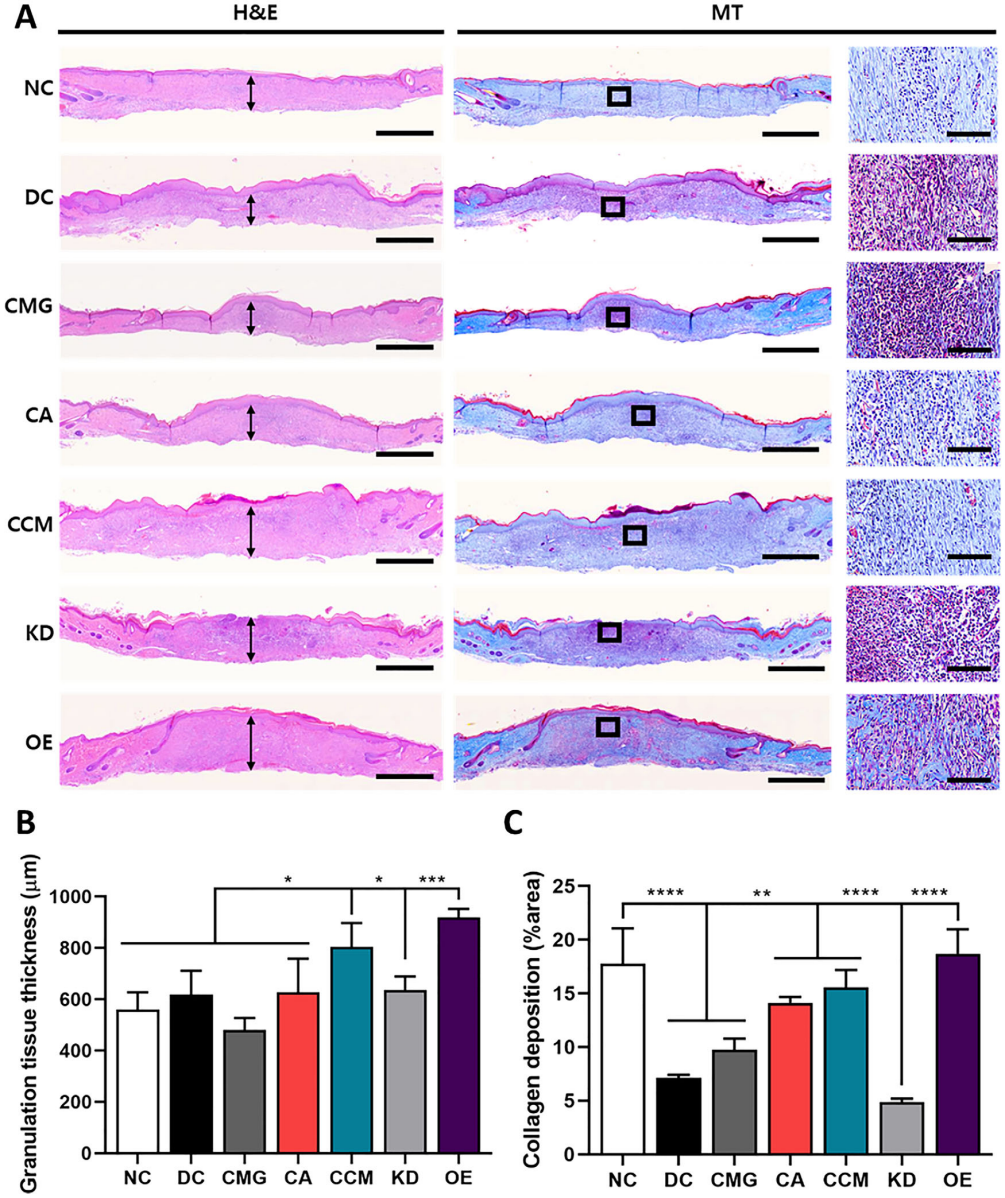
Histological analysis of skin harvested from the rat DFU model. A) Representative images of H&E‐ and MT‐stained skin tissues from NC, DC, CMG, CA, CCM, KD, and OE groups at 21 days. Scale bars: 1 mm, inset: 100 µm. Black arrows indicate granulation tissue thickness. B,C) Quantification of granulation tissue thickness B) and collagen deposition C) in skin tissues from each treatment group at 21 days, measured using ImageJ (*n* = 5 per group). All data are presented as mean ± SD. One‐way ANOVA; ^*^ *p* < 0.05, ^***^ *p* < 0.001, ^****^ *p* < 0.0001.

**Figure 6:**
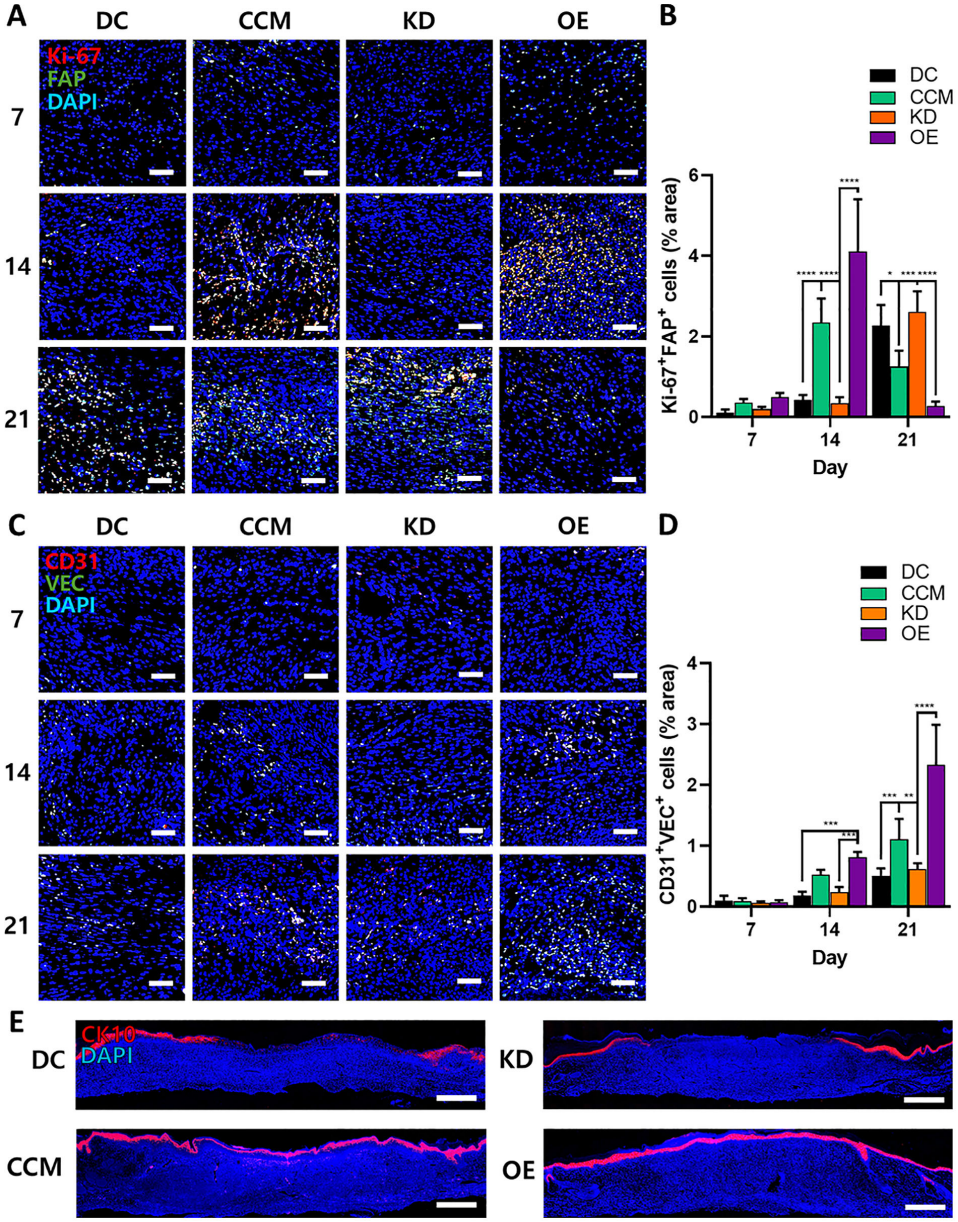
Immunofluorescent analysis of the wound healing process. A,C,E) Representative fluorescent images of skin tissue samples harvested from DC, CCM, KD, and OE groups at 7, 14, and 21 days. Samples were stained for Ki‐67 and FAP (A), CD31 and VEC (C), or CK10 (E) to evaluate the effect of IL‐8 on wound healing in the DFU rat model. Nuclei were counterstained with DAPI. Scale bars: 50 µm (A,C) and 500 µm (E). B,D) Quantification of Ki‐67^+^FAP^+^ area (B) and CD31^+^VEC^+^ area (D) in skin tissue samples from all treatment groups at 7, 14, and 21 days, as determined using ImageJ (*n* = 5 per group). All data are presented as mean ± SD. One‐way ANOVA; ^*^ *p* < 0.05, ^**^ *p* < 0.01, ^***^ *p* < 0.001, ^****^ *p* < 0.0001.

Histological analysis showed that CMG alone produced no significant increase in granulation thickness compared with the DC group, whereas CCMs significantly enhanced granulation and collagen deposition, which were reduced in the KD group and further enhanced in the OE group (**Figure** **5**A–C). Immunofluorescence staining for Ki‐67 and fibroblast activation protein (FAP) demonstrated elevated proliferative activity in the CCM and OE groups on day 14, with reduced levels in the KD group. By day 21, Ki‐67^+^FAP^+^ areas decreased in CCM and OE groups, suggesting progression toward the resolution of the proliferative phase (**Figure** **6**A,B; Figure S5A, Supporting Information). Staining for cluster of differentiation 31 (CD31) and vascular endothelial cadherin (VEC) revealed increased angiogenesis in the CCM and OE groups, whereas vascularization was attenuated in the KD group (Figure 6C,D; Figure S5B, Supporting Information). Cytokeratin 10 (CK10) staining confirmed complete epidermal restoration in the OE group, near‐complete regeneration in the CCM group, and incomplete re‐epithelialization in the other groups (Figure 6E; Figure S5C, Supporting Information). These findings indicate the essential role of IL‐8 in angiogenesis and epidermal regeneration.

Collectively, these comprehensive results established the cell:CMG ratio as a critical determinant of microtissue functionality and identified IL‐8 expression—regulated through FGFR‐integrin‐ERK signaling—as a key modulator of cell survival and regenerative outcomes. IL‐8 overexpression within the CCM platform markedly enhances cell‐mediated migration, proliferation, angiogenesis, and wound healing, highlighting IL‐8 as a promising therapeutic target for DFU treatment.

## 3 Discussion

Stem cell therapy holds significant promise for tissue regeneration across diverse clinical applications. However, its therapeutic potential remains limited by poor survival and low engraftment of transplanted cells. As high cell viability is essential for sustained paracrine factor secretion—a key mechanism in stem cell‐mediated tissue repair—strategies to enhance survival are critically needed. In this study, we demonstrated that the CMG platform provides a microporous microarchitecture that enhances cell adhesion, suppresses apoptosis, and supports long‐term viability of hASCs. Notably, CCMs exhibited elevated IL‐8 expression mediated through FGFR‐ and integrin‐dependent ERK signaling. Given the well‐documented roles of IL‐8 in promoting angiogenesis, cell migration, and proliferation, we identified it as a therapeutic target to enhance diabetic wound healing. To further improve therapeutic efficacy, we genetically modified hASCs using an adenoviral vector to overexpress IL‐8 and assembled them into CCMs, generating IL‐8‐overexpressing CCMs. This approach effectively accelerated wound healing in a rat DFU model.

The microgel‐based scaffold provided a favorable microporous microenvironment that supported mass transfer, cell adhesion, and survival—features essential for tissue engineering. Our findings indicate that the cell:CMG ratio is a critical determinant of microtissue properties, affecting porosity, FA formation, viability, and gene expression. Integrin‐mediated adhesion was shown to be highly sensitive to the density of adhesion sites. A cell:CMG ratio of 1:4 yielded optimal adhesion, as evidenced by increased pFAK levels, larger FA area, improved mechanical properties, and minimal apoptosis. In contrast, excessively high CMG content (ratios ≥ 1:8) impaired adhesion, likely due to reduced cell‐mediated cross‐linking and poor structural integrity (Figure 1C–E; Figure S1B,E, Supporting Information). These findings align with previous reports showing that integrin‐mediated adhesion depends on the density of available adhesion sites, with higher adhesion site density leading to increased FA number, area, and stability,^[^ ^35^ ^]^ whereas excessive microgel density can restrict cell spreading and limit adhesion.^[^ ^36^ ^]^ Given that stable FAs activate signaling pathways essential for cell survival and that loss of adhesion can induce anoikis,^[^ ^37^ ^]^ we evaluated cell viability over 21 days. Consistently, the 1:4 ratio exhibited the lowest cleaved caspase‐3 levels and maintained the highest cell survival throughout the culture (Figure 1D–H; Figure S1F, Supporting Information).

IL‐8 was significantly upregulated in CCMs compared with CAs. This upregulation was attributed to microenvironmental factors such as hypoxia and 3D architecture, as well as biochemical cues from basic fibroblast growth factor (bFGF) and collagen‐integrin interactions. Mechanistic studies revealed that FGFR signaling was the primary regulator of IL‐8 expression in CAs, whereas IL‐8 expression in CCMs was co‐regulated by FGFR, integrin, and HIF1α pathways, with FGFR and integrin playing dominant roles (Figure 2A–G). FGFRs and integrins share downstream effectors such as the Ras/MAPK and PI3K/Akt pathways.^[^ ^38^, ^39^ ^]^ Downstream, ERK and p38 signaling served as the principal effectors in CAs, with JNK contributing to a lesser extent. In CCMs, IL‐8 expression was predominantly dependent on ERK signaling. Although previous studies have suggested potential crosstalk between integrins and growth factor receptors, including FGFRs,^[^ ^40^ ^]^ the precise mechanisms of this interaction in IL‐8 regulation remain to be elucidated. Our findings support a model in which IL‐8 upregulation in CCMs is primarily mediated by integrin and FGFR signaling that converge on the ERK pathway.

While collagen‐based biomaterials are known to upregulate regenerative cytokines, our previous work showed that IL‐8 secretion from CCMs is transient.^[^ ^16^ ^]^ To sustain elevated IL‐8 expression, we employed adenoviral transduction. Genetic modification represents a feasible strategy for achieving prolonged therapeutic gene expression.^[^ ^41^, ^42^, ^43^ ^]^ In vitro, IL‐8 overexpression enhanced keratinocyte and fibroblast proliferation and migration, supporting its role in promoting wound closure and granulation tissue formation.

Although the pro‐inflammatory phase is an essential component of wound healing, failure to transition to the anti‐inflammatory phase can result in chronic inflammation and delayed tissue repair. IL‐8, a potent pro‐inflammatory chemokine, plays a critical role in recruiting neutrophils to injury sites, thereby facilitating debris clearance and infection control. Elevated IL‐8 expression has been consistently reported in various chronic inflammatory conditions, including psoriasis, chronic obstructive pulmonary disease, rheumatoid arthritis, and nonhealing wounds, with IL‐8 levels correlating with disease severity.^[^ ^44^, ^45^ ^]^ Notably, IL‐8 receptor beta (IL‐8RB) expression is markedly reduced in chronic wounds compared with acute wounds, which may partly explain the elevated IL‐8 levels observed in chronic inflammation.^[^ ^46^ ^]^ Functional studies have demonstrated that IL‐8 is essential for normal wound healing, as IL‐8RB knockout mice exhibit delayed wound closure, whereas topical IL‐8 application accelerates keratinocyte proliferation and re‐epithelialization without inducing adverse inflammatory effects.^[^ ^21^, ^47^ ^]^ Moreover, although excessive IL‐8 administration can enhance neutrophil recruitment, in vivo studies have shown no evidence of exacerbated chronic inflammation. For example, recombinant IL‐8 administration in large animals increased neutrophil infiltration while reducing uterine inflammation.^[^ ^48^ ^]^ Similarly, intravenous administration of radiolabeled IL‐8 led to selective accumulation at infection sites without affecting peripheral lymphocyte populations.^[^ ^49^ ^]^ Collectively, these findings suggest that the immunomodulatory effects of IL‐8 are highly context‐dependent. While elevated IL‐8 is often associated with chronic inflammation, exogenous IL‐8 administration appears to enhance the local innate immune responses without causing deleterious immune dysregulation. Nevertheless, disentangling the causal role of IL‐8 in inflammation remains challenging, as chemokine‐driven immune responses are highly complex and interdependent on other inflammatory mediators.

Injectable hydrogels^[^ ^32^ ^]^ enable minimally invasive delivery but degrade rapidly; organoid systems^[^ ^50^, ^51^ ^]^ replicate physiological complexity but face scalability issues; and extracellular vesicle‐based therapies offer low immunogenicity yet exhibit short retention. CCMs combine the injectability of viable hASCs with the structural and biological benefits of CMG, bridging acellular and cell‐based strategies to provide a balanced translational platform.

Compared with gelatin hydrogels,^[^ ^52^ ^]^ poly(lactic‐co‐glycolic acid) matrices,^[^ ^53^ ^]^ and electrospun polycaprolactone scaffolds^[^ ^54^ ^]^—which are limited by rapid degradation, acidic byproducts,^[^ ^55^ ^]^ or insufficient pore interconnectivity—CMG scaffolds maintain a stable, biocompatible, and microporous environment that supports long‐term cell survival and sustained cytokine secretion. The porous structure enhances oxygen and nutrient diffusion while promoting integrin‐collagen interactions, reducing apoptosis, and stabilizing cell‐matrix adhesion.^[^ ^16^, ^56^ ^]^ These features collectively enable sustained paracrine signaling and superior regenerative outcomes.

This study demonstrates that combining CMG scaffolds with IL‐8 overexpression in hASCs provides a robust platform to enhance stem cell viability, function, and therapeutic outcomes, particularly for diabetic wound treatment. Building on these significant therapeutic outcomes in the DFU model, we believe that further refinement of the CMG platform may facilitate successful clinical translation and expand its applicability to other disease models. For instance, incorporating cross‐linkers to enhance mechanical properties and control degradation rates could further sustain cell survival and bioactivity post‐transplantation. Co‐overexpression of IL‐8 with factors such as bFGF, hepatocyte growth factor (HGF), and vascular endothelial growth factor (VEGF) could further amplify angiogenic and tissue‐regenerative responses. These growth factors co‐activate the PI3K/Akt and ERK1/2 pathways,^[^ ^28^, ^57^ ^]^ thereby promoting angiogenesis, proliferation, and matrix remodeling. IL‐8 has been shown to upregulate VEGF via PI3K/Akt and MAPK/ERK signaling and through VEGF receptor transactivation^[^ ^57^, ^58^ ^]^ while HGF and TIMP1 enhance cytoprotection and ECM remodeling.^[^ ^59^, ^60^ ^]^ Such combined expression may initiate coordinated inflammation, angiogenesis, and remodeling, thereby enhancing wound repair. Collectively, these strategies highlight the potential of optimized CMG scaffolds with targeted gene overexpression as a versatile and powerful platform for the treatment of diabetic wounds and other pathological disorders.

## 4 Conclusion

This study underscores the efficacy of the CMG‐based platform in supporting stem cell survival, adhesion, and function—key parameters for effective tissue regeneration. Notably, an optimal cell:CMG ratio of 1:4 was identified to maximize adhesion, viability, and cytokine secretion. IL‐8, a cytokine with well‐established angiogenic, proliferative, and migratory functions, was identified as a key mediator of wound healing within the diabetic microenvironment. By genetically modifying hASCs to overexpress IL‐8 and incorporating them into CMG scaffolds, we developed CCMs that exhibited superior therapeutic efficacy in both in vitro assays and in vivo DFU models. IL‐8 overexpression markedly enhanced keratinocyte and fibroblast activity, whereas its knockdown impaired these effects, highlighting the central role of IL‐8 in regenerative processes. Collectively, these findings demonstrate that the strategic combination of engineered scaffolds with targeted gene modulation offers a promising and versatile platform for enhancing stem cell‐based therapies in chronic wound treatment and potentially other regenerative applications.

## 5 Experimental Section

### Materials

The optimal concentrations for siRNA against HIF1α, FGFR, and ITGB1 were predetermined as 10 nm (Figure S2A–C, Supporting Information) and purchased from Santa Cruz Biotechnology (TX, USA). The optimal concentrations for chemical inhibitors targeting HIF1α (KC7F2, Selleck Chemicals, TX, USA), FGFR (PD173074, Tocris Bioscience, Bristol, UK) and function‐blocking antibody against ITGB1 (Cat. 552828, BD Biosciences, CA, USA) were established as 20 µm, 40 nm and 5 µg mL^−1^, respectively (Figure S2D–I, Supporting Information). Chemical inhibitors targeting Akt (LY294002), ERK (U0126), JNK (SP600125), and p38 (SB203580) were used at 10 µm (Sigma–Aldrich, MO, USA).

### Collagen Microgel Fabrication

CMGs were fabricated following the method described by Chung et al.^[^ ^16^ ^]^ Atelocollagen type I derived from porcine skin (white, sponge form; purity confirmed by SDS‐PAGE) was purchased from MSBio, Inc. (Seongnam, South Korea). Sodium hyaluronate (white powder; ≥ 95% purity; intrinsic viscosity 0.79 m^3^ kg^−1^) was purchased from Contipro Inc. (Dolní Dobrouč, Czech Republic). To prepare the initial stock solutions, atelocollagen was dissolved in 0.01 N hydrochloric acid to obtain a 25% (w/w) collagen stock solution, and sodium hyaluronate was dissolved in sterile PBS to obtain 10% (w/w) hyaluronic acid (HA) stock solution. To form the hydrogel composite, the collagen and HA stock solutions were combined to achieve final concentrations of 10% (w/w) collagen and 5% (w/w) HA. The neutralized hydrogel composite was then incubated at 37 °C to induce gelation. The resulting hydrogel was mechanically disrupted in culture medium using a bead beater and filtered through a 100 µm filter.

### Cell Culture

Human adipose‐derived stem cells (S. Biomedics, Seoul, South Korea) were expanded up to passage 5 using CEFOgro medium (CEFO Co., Seoul, South Korea) and cultured in an incubator maintained at 37 °C and 5% CO_2_. At passage 5, hASCs were resuspended in serum‐free medium (SFM, Gibco, Waltham, MA, USA) and seeded into 96‐well round‐bottom ultralow attachment plates at 2 × 10^5^ cells per well with cell:CMG ratio of 1:0, 1:2, 1:4, 1:8, or 1:16.

### Rheology

To assess the rheological properties of 3D constructs, hASCs were cultured with CMGs at cell:CMG ratios of 1:2, 1:4, 1:8, or 1:16. Samples were measured using an MCR 102 rheometer (Anton‐Paar, Graz, Austria) under dynamic time sweep testing at a frequency of 10 rad s^−1^ and 0.5% strain at 25 °C until a plateau was reached.

### Cryo‐SEM

Samples prepared at cell:CMG ratios of 1:0, 1:2, 1:4, 1:8, 1:16 were mounted on slit holders and snap‐frozen in liquid nitrogen. The samples were fractured with a cold blade to expose the inner microstructure and then transferred to a cryo‐stage for imaging. Cryo‐SEM images were acquired using a Quanta 3D focused ion beam scanning electron microscope (FEI, Hillsboro, OR, USA).

### LIVE/DEAD

LIVE/DEAD staining of 3D constructs was performed by incubating the samples with 20 µm calcein AM and 10 µm propidium iodide at 37 °C for 5 h. Samples were then fixed, cryosectioned into 10 µm‐thick slices, and imaged under a confocal microscope (LSM 700, Zeiss, Oberkochen, Germany).

### Real‐Time Polymerase Chain Reaction (RT‐PCR)

The mRNA extraction was performed using the cetyltrimethylammonium bromide (CTAB) method described by Wang et al.^[^ ^61^ ^]^ Briefly, samples were mixed with CTAB buffer containing 1% beta‐mercaptoethanol at 65 °C and extracted twice with an equal volume of chloroform. The upper phase was collected, precipitated with isopropanol, and centrifuged. The supernatant was discarded, and the pellet was washed with 75% ethanol and dissolved in RNase‐free water. The RNA was further purified using the Qiagen RNeasy Mini kit according to the manufacturer's protocol. The sequence of primers used in this study is listed in Table S1 (Supporting Information).

### Enzyme Linked Immunosorbent Assay (ELISA)

Conditioned media were collected by incubating 3D constructs in SFM without supplements for 24 h. Quantification of growth factors was performed using ELISA kits (R&D Systems, Minneapolis, MN, USA; RayBiotech, Peachtree Corners, GA, USA) according to the manufacturers’ instructions.

### Western Blot

Proteins were isolated from CCM following the procedure outlined by André et al.^[^ ^62^ ^]^ Isolated proteins were resuspended in lysis buffer (100 mm Tris pH 7.2, 20 mm EDTA, 140 mm NaCl, 5% SDS) by sonication and incubated at 50 °C for 1 h for optimal solubilization as described by Kopec et al.^[^ ^63^ ^]^ Protein concentrations were determined using a bicinchoninic acid (BCA) assay. Lysates were subjected to SDS‐PAGE using a 4–15% gradient gel (Bio‐Rad Laboratories, Hercules, CA, USA) and transferred to polyvinylidene fluoride membranes (Millipore, Burlington, MA, USA). Membranes were blocked in West‐Ezier super blocking buffer (GenDEPOT, Katy, TX, USA) and incubated with primary and secondary antibodies at 4 °C at the dilutions specified in Table S2 (Supporting Information). Detection was performed using an iBright CL1500 chemiluminescence imaging system (Thermo Fisher Scientific).

### IL‐8 Knockdown and Overexpression

shRNA targeting the human IL‐8 gene was purchased from Genecopoeia (Rockville, MD, USA). Recombinant adenovirus (rAd) encoding the human IL‐8 gene was purchased from Koma Biotech (Seoul, South Korea). Cells were seeded at 1 x 10^5^ cells per well in 6‐well plates and cultured for 24 h. For IL‐8 knockdown, cells were transfected with shRNA using Lipofectamine 3000 (Invitrogen) according to the manufacturer's instructions. For IL‐8 overexpression, cells were treated with rAd for 3 h and subsequently cultured in fresh medium overnight. The optimal concentrations for shRNA and rAd were predetermined to be 0.01 µg mL^−1^ and 300 multiplicity of infection (MOI), respectively (Figure S2A–D, Supporting Information).

### Tube Formation Assay

GFP‐tagged HUVECs were purchased from Lonza (Basel, Switzerland). Growth factor‐reduced basement membrane extract (Matrigel, Corning, Corning, NY, USA) was added to the lower compartment of 24‐well transwell plates, and serum‐starved HUVECs were seeded at 1 × 10^5^ cells per well. To the upper compartment, endothelial basal medium (EBM), endothelial growth medium (EGM), CMG, CA, CCM, KD, or OE were added and co‐cultured with HUVECs for 16 h. Images were acquired using a confocal microscope and analyzed with ImageJ.

### Scratch Assay

Human dermal fibroblasts (CEFOBio, Seoul, South Korea) or human epidermal keratinocytes (HaCaT) were seeded in the lower compartment of 24‐well transwell plates at 5 × 10^4^ cells per well and cultured for 24 h. A scratch was created at the center of each well using a 200 µL pipette tip. Wells were washed once with PBS and co‐cultured with control, CMG, CA, CCM, KD, or OE in the upper compartment in serum‐free DMEM for 24 h. Images were obtained using an Axio Observer inverted microscope (Zeiss, Oberkochen, Germany).

### Immunofluorescence Assay

Cryosections or paraffin sections (10 µm thick) were permeabilized with 0.5% Triton X‐100 and blocked with 2% BSA. Sections were incubated with primary antibodies at 4 °C at the dilutions specified in Table S2 (Supporting Information), followed by Alexa Fluor‐conjugated secondary antibodies (1:500) for 1 h at room temperature. After washing, sections were mounted with Vectashield mounting medium (Vector Laboratories, Newark, CA, USA) and coverslipped. Images were acquired using a confocal microscope and analyzed with ImageJ software.

### Establishment of Diabetic Foot Ulcer Model

All animal experiments were performed in accordance with the guidelines of the National Institutes of Health of South Korea and were approved by the International Animal Care and Use Committee of the Korea Institute of Science and Technology [KIST‐IACUC‐2023‐034‐2]. Rats had ad libitum access to food and water and were housed in a facility maintained at 24 °C, 60% humidity, and a 12‐h light/dark cycle. Eight‐week‐old male Sprague‐Dawley rats were purchased from DBL Co., Ltd. (Eumseong, South Korea) and acclimated for at least 1 week. Diabetes was induced by intraperitoneal injection of streptozotocin (70 mg kg^−1^). One week later, rats with blood glucose levels > 300 mg dL^−1^ were considered diabetic (Table S3, Supporting Information). To establish the DFU model, rats were anesthetized with isoflurane, and a single full‐thickness circular wound was created on the dorsal surface of the right hind foot using a 6 mm biopsy punch and Westcott scissors. Photographs were captured on days 0, 7, 14, and 21 post‐surgery, and the relative wound area was calculated using the following formula:

(1) $$\mathrm{Relative}\;\mathrm{wound}\;\mathrm{area}\;\left(\%\right)=\left(A_{t}/A_{0}\times 100\right)$$

where A_t_ represents the wound area at each time point (day 0, 7, 14, or 21), and A_0_ represents the initial wound area on day 0.

### Statistical Analysis

All statistical analyses were conducted using Prism 7 software (GraphPad, San Diego, CA, USA). Data were presented as mean ± standard deviation (SD). Comparisons among multiple groups were performed using one‐way or two‐way ANOVA under the assumption of normal distribution and equal variance, followed by post hoc multiple comparison tests. A p‐value of less than 0.05 was considered statistically significant.

### Ethical Approval

All animal experiments were performed in accordance with the guidelines of the National Institutes of Health of South Korea and approved by the Institutional Animal Care and Use Committee of the Korea Institute of Science and Technology [KIST‐IACUC‐2023‐034‐2].

## Conflict of Interest

The authors declare no conflict of interest.

## Author Contributions

H.C. and W.Y.J. contributed equally to this work. H.C. contributed to conceptualization, methodology, investigation, formal analysis, writing—original draft, writing—review and editing, and visualization. W.Y.J. contributed to conceptualization, methodology, investigation, formal analysis, and writing—review and editing. J.‐K.C. contributed to methodology, formal analysis, and writing—review and editing, and S.‐H.K. contributed to conceptualization, methodology, writing—original draft, writing—review and editing, supervision, project administration, and funding acquisition.

## Supporting information



Supporting Information

## Data Availability

All data presented and/or analyzed in this paper are available from the corresponding author upon reasonable request.
