# Supplementary material for: Interleukin‐8 Overexpressing Collagen Microgel‐Based Cellular Microtissue Accelerates the Healing of Diabetic Foot Ulcers
Source: Small. 2025 Nov 26;22(3):e11027. doi: 10.1002/smll.202511027 (PMC12802542; doi:10.1002/smll.202511027)
Supplement: Supplementary file 1 — Supporting Information [file SMLL-22-e11027-s001.docx]

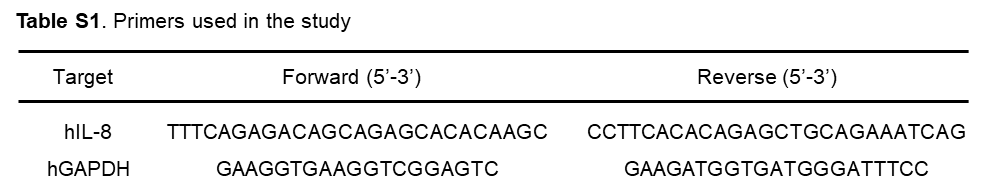


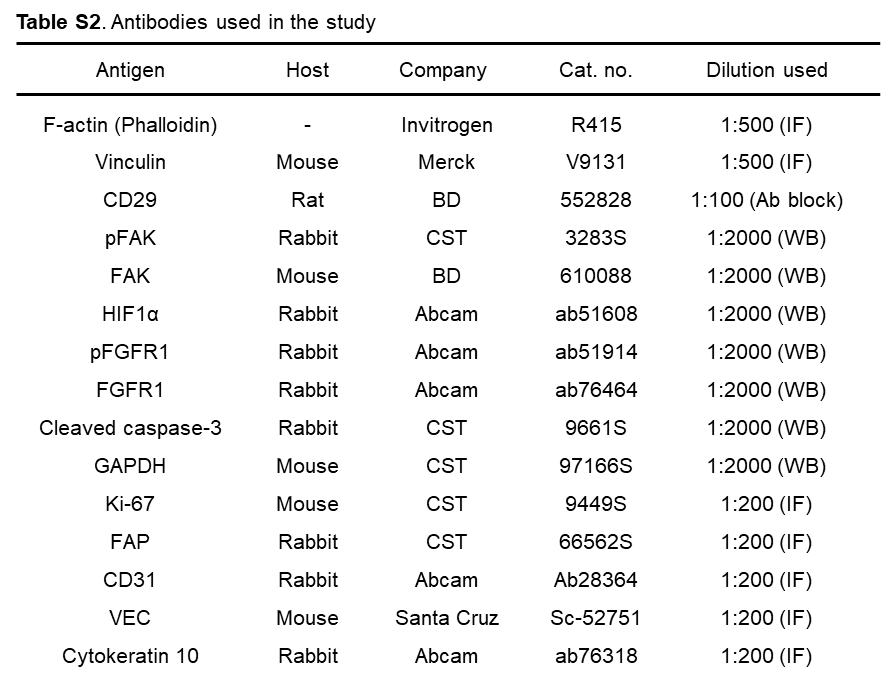


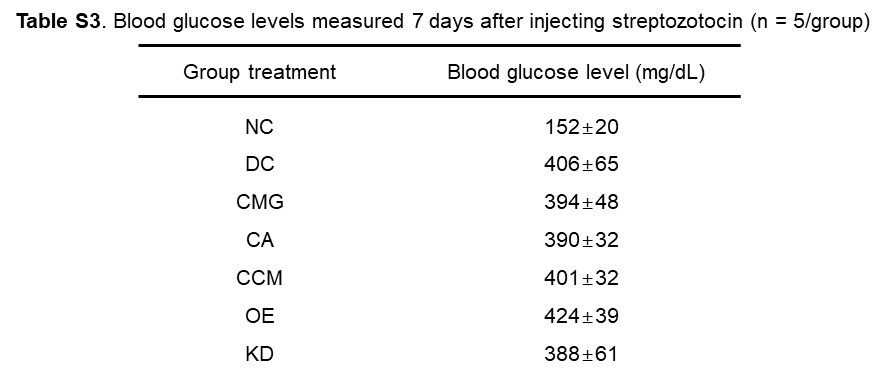


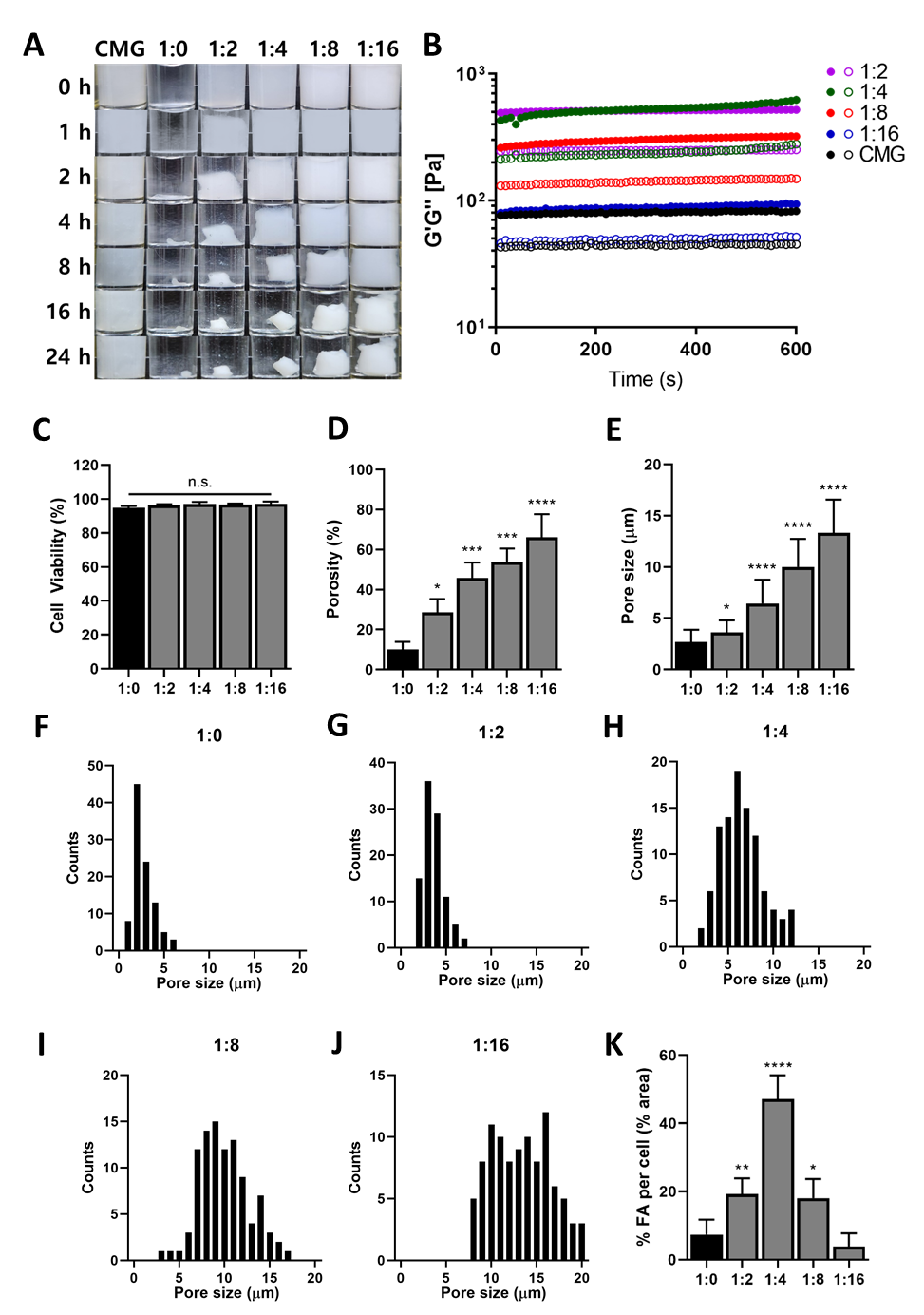


**Figure S1. Effect of varying cell:CMG ratio.** (A) Representative digital photographs showing 3D construct formation after culturing hASCs with CMG at varying ratios (1:0, 1:2, 1:4, 1:8, and 1:16; cell:CMG ratio by pellet volume, 2 × 10^5^ hASCs/sample) for 24 h (B) Rheological properties of CMG and 3D cell constructs. Storage moduli is represented by solid circles and loss moduli by open circles. (C) Quantification of cell viability of 3D cell constructs at 1 d as determined by trypan blue exclusion assay (n=3/group). (D-J) Quantification of porosity (D), average pore size (E) and pore size distribution at ratios of 1:0 (F), 1:2 (G), 1:4 (H), 1:8 (I) and 1:16 (J), as analyzed from cryo-SEM images using ImageJ (n=3/group for panel D; n=100/group for panels E-J). (K) Quantification of percent focal adhesion area per cell, determined using ImageJ (n=5/group). All data are presented as mean ± SD. One-way ANOVA; n.s., not significant; * p < 0.05, ** p < 0.01, *** p < 0.001, **** p < 0.0001 compared with the 1:0 group.


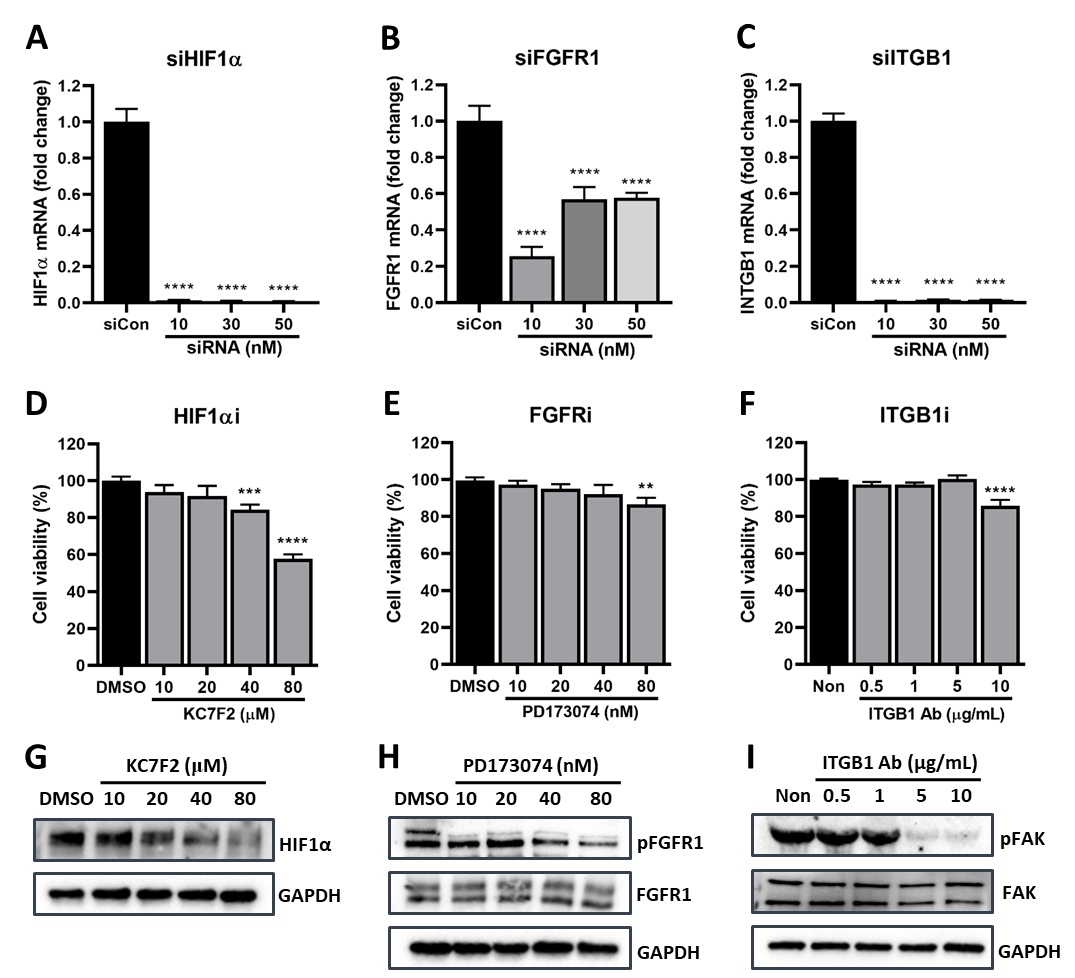


**Figure S2. Optimization of siRNA and inhibitor concentrations.** (A-C) Quantification of mRNA expression levels of HIF1α (A), FGFR1 (B), and ITGB1(C) following siRNA-mediated knockdown of target genes, as determined by RT-PCR (n=3/group). One-way ANOVA; **** p < 0.0001 compared to siCon group. (D-F) Cell viability of CCMs after treatment with inhibitors against HIF1α (KC7F2; D) and FGFR (PD173074; E), and a function-blocking antibody against ITGB1 (F), as determined by the trypan blue exclusion assay (n=3/group). One-way ANOVA; ** p < 0.01, *** p < 0.001, **** p < 0.0001 compared with the DMSO group. All data are presented as mean ± SD. (G-I) Western blot analysis of CCMs treated with inhibitors against HIF1α (G) and FGFR1 (H), and a function-blocking antibody against ITGB1(I), confirming target inhibition. GAPDH was used as loading control.


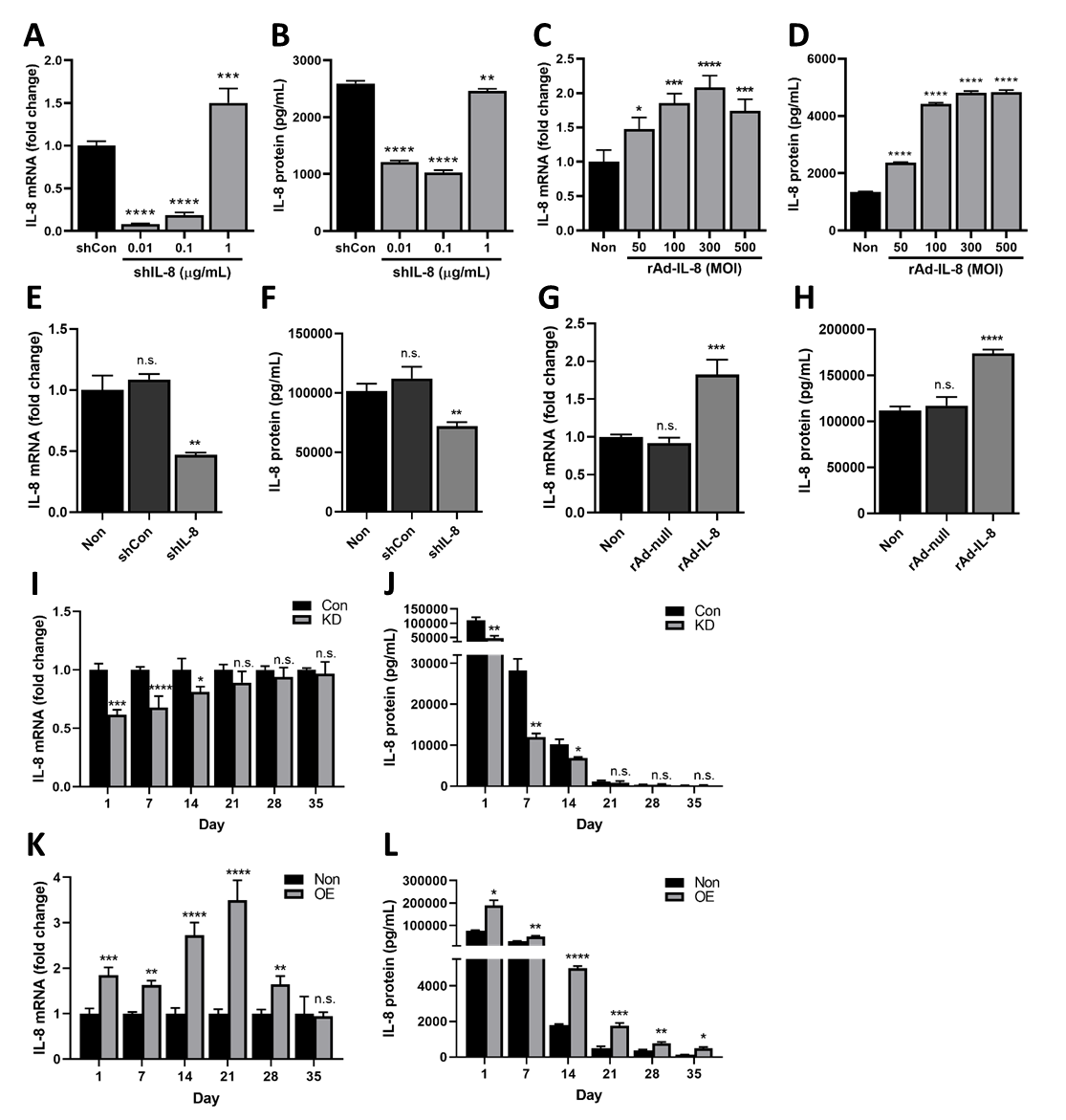


**Figure S3. IL-8 knockdown and overexpression via shRNA transfection and adenoviral transduction.** (A-B) IL-8 expression at the mRNA (A) and protein (B) levels in hASCs grown in monolayer following transfection with shRNA targeting IL-8 (shIL-8) at varying concentrations (n=3/group). (C-D) IL-8 expression at the mRNA (C) and protein (D) levels in hASCs grown in monolayer following transduction with recombinant adenovirus encoding IL-8 (rAd-IL-8) at varying multiplicities of infection (MOI) (n=3/group). (E-F) IL-8 expression at the mRNA (E) and protein (F) levels in CCMs after transfection with shIL-8 at 0.01 μg/mL (n=3/group). (G-H) IL-8 expression at the mRNA (G) and protein (H) levels in CCMs after transduction with rAd-IL-8 at 300 MOI (n=3/group). (I-J) Time-course analysis of IL-8 knockdown at the mRNA (I) and protein (J) levels after shIL-8 transfection over 35 d (n=3/group). (K-L) Time-course analysis of IL-8 overexpression at the mRNA (K) and protein (L) levels after rAd-IL-8 transduction over 35 d (n=3/group). All data are presented as mean ± SD. One-way ANOVA; n.s., not significant; * p < 0.05, ** p < 0.01, *** p < 0.001, **** p < 0.0001 compared with the control group.


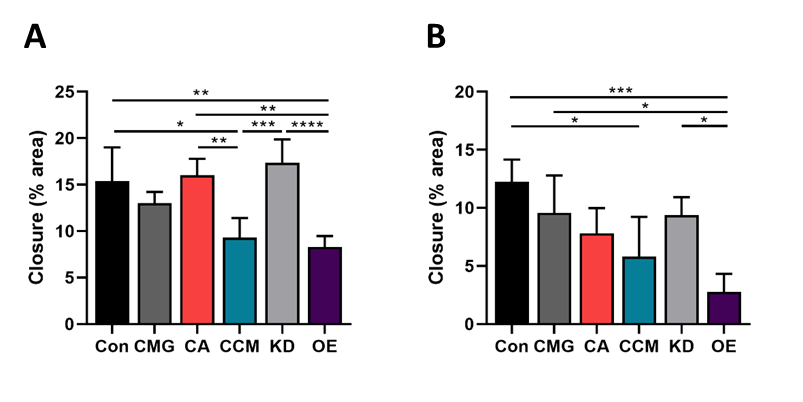


**Figure S4. Effect of IL-8 on the migration of keratinocytes and fibroblasts.** (A-B) Quantification of scratch wound closure by keratinocytes (A) and fibroblasts (B) at 24 h after co-culture with Con, CMG, CA, CCM, KD, and OE groups, as analyzed using ImageJ (n=5/group). All data are presented as mean ± SD. One-way ANOVA; * p < 0.05, ** p < 0.01, *** p < 0.001, **** p < 0.0001 compared with the control group.


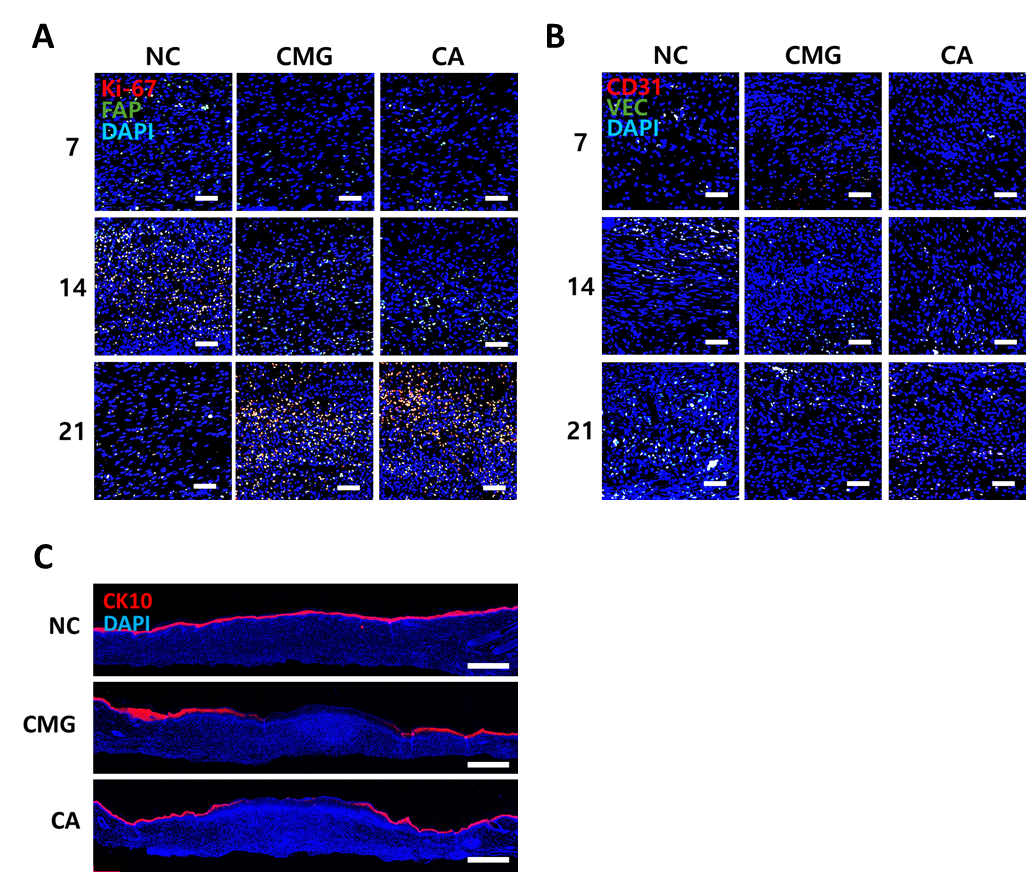


**Figure S5. Immunofluorescent analysis of wound healing process in control groups.** (A-C) Representative fluorescent images of skin tissue samples harvested from NC, CMG, and CA groups at 7, 14, and 21 d. Samples were stained for Ki-67 and FAP (A), CD31 and VEC (B), or CK10 (C) to analyze the wound healing process in the DFU rat model. DAPI was used for nuclear staining. Scale bars: 50 μm (A, B) and 500 μm (C).
